# The New Medical Bill

**Published:** 1883-12

**Authors:** 


### Editorial.

### THE NEW MEDICAL BILL.

We are glad to be able to announce that the committee having this important act of the Erie County Society in charge are daily receiving evidence that the admirable provisions of the bill appeal to the common sense of the community, and is thereby receiving a cordial co-operation, both from the profession and the laity, as we anticipated. Dr. Morris' letter, as published in our last number, has called forth much favorable comment. The *New York Medical Journal*, in an editorial upon the bill, objects to allowing the Homœopaths two members and the Eclectics one, asserting that a representation of one to each of the sects would satisfy all the reasonable claims of each of these sections of the profession. It further says that "an outcry against the bill must be expected from a certain class of doctors—a class always willing to pose as victims to persecution; but they will oppose any reasonable bill, because being their only idea of liberty. Dealing with sensible men, as most of our legislators may be assumed to be, there ought to be little difficulty in showing the utter shallowness of the persecution plea." We do not agree with our contemporary as to the wisdom of its proposed change as to representation. "We do not ask for legislation in the interests of the medical profession, but for measures in the interests of the community;" and it is only right and justice that in seeking that we should show a disposition to give all legally qualified medical men all that they can reasonably ask in their representation on the proposed Board; and if the Eclectics are entitled to one representative, then the Homœopaths can fairly claim two, and, as we know, will not be content with less. That point granted, there appears to be an almost unanimous feeling among the educated men in favor of the bill.

The committee have recently issued a memorial to the profession of the State of New York, which contains so many facts and arguments in favor of the bill that we reproduce its eleven propositions entire:

1. That the present system of licensing physicians does not command the respect and confidence of either the profession or the people.

2. That the cardinal defect in that system is the fact that diplomas of medical colleges are licenses to practice medicine.

3. That, as a general rule, medical colleges are private corporations, operated by, and in the interests of, a few individuals, whose personal and pecuniary interests—acting with the tendencies of competition—have brought about a state of affairs in which a diploma has practically ceased to furnish any reasonable presumption of respectable professonal acquirement.

4. That the graduates from the medical schools of this country, during the year last past, were nearly four thousand in number (3,979), being more than the number graduated during the same period by all the medical schools of the whole British Empire, France, Austria and Germany combined.

5. That, as a result of this system, the country is overrun with unlearned, incompetent persons, holding licenses to practice medicine, to the serious degradation of the profession and the detriment of the public health and welfare.

6. That the existence of this evil has recently been recognized by the legislative authority of the States of Alabama, North Carolina, Illinois, Minnesota, Missouri and West Virginia, and the remedy applied by the establishment, in each of these commonwealths, of a single licensing body after the form and manner of the faculty which this act would create.

7. That this system of placing the licensing power in the hands of a body directly responsible to the people, and entirely independent of, and disconnected from the teaching bodies, is the one which prevails in almost every civilized country in the world.

8. That the object of this proposed law is to strike at ignorance and incompetency, but that it in no way interferes with freedom of opinion or individuality of doctrine.

9. That, if enacted, it will in no way interfere with any person now engaged in the lawful practice of medicine, inasmuch as it excludes all such persons from its operations.

10. That the proposed law involves the State in no expenditure of money, neither now nor hereafter.

11. That, inasmuch as the evil complained of in this memorial is conceded by all, save those interested in its perpetuation—and as the plan here proposed is now in successful operation in six different States—inasmuch as this plan will not disturb any person now engaged in the lawful practice of medicine, and as it works no wrong to any citizen of the State—inasmuch as it imposes no burdens upon the State treasury, and would be simply adopting the usage of other enlightened countries—and inasmuch as its sole object is to secure to the people the services of a worthy and efficient medical profession, in the belief that such services would promote the public health and public welfare—for these reasons and in the name and behalf of the Medical Society of the County of Erie, we ask your hearty co-operation in the effort to secure the passage of this measure.

It is highly expedient that the attention of each member of the legislature be suitably directed to these facts, and we hope that each one of our many readers in the State of New York will personally see to it that the representative of his section understands the provisions of the bill, and so be prepared to support it in spite of all the efforts which colleges and quacks may make to kill it.

